# Genotypic, Phenotypic and Clinical Validation of GeneXpert in Extra-Pulmonary and Pulmonary Tuberculosis in India

**DOI:** 10.1371/journal.pone.0149258

**Published:** 2016-02-19

**Authors:** Urvashi B. Singh, Pooja Pandey, Girija Mehta, Anuj K. Bhatnagar, Anant Mohan, Vinay Goyal, Vineet Ahuja, Ranjani Ramachandran, Kuldeep S. Sachdeva, Jyotish C. Samantaray

**Affiliations:** 1 Department of Microbiology, All India Institute of Medical Sciences, New Delhi, India; 2 Rajan Babu Institute for Pulmonary Medicine and Tuberculosis, Delhi, India; 3 Department of Pulmonary Medicine and Sleep Disorders, All India Institute of Medical Sciences, New Delhi, India; 4 Department of Neurology, All India Institute of Medical Sciences, New Delhi, India; 5 Department of Gastroenterology and Human Nutrition, All India Institute of Medical Sciences, New Delhi, India; 6 WHO, Country Office for India, New Delhi, India; 7 Central TB Division, Government of India, New Delhi, India; Barcelona University Hospital, SPAIN

## Abstract

**Background:**

Newer molecular diagnostics have brought paradigm shift in early diagnosis of tuberculosis [TB]. WHO recommended use of GeneXpert MTB/RIF [Xpert] for Extra-pulmonary [EP] TB; critics have since questioned its efficiency.

**Methods:**

The present study was designed to assess the performance of GeneXpert in 761 extra-pulmonary and 384 pulmonary specimens from patients clinically suspected of TB and compare with Phenotypic, Genotypic and Composite reference standards [CRS].

**Results:**

Comparison of GeneXpert results to CRS, demonstrated sensitivity of 100% and 90.68%, specificity of 100% and 99.62% for pulmonary and extra-pulmonary samples. On comparison with culture, sensitivity for Rifampicin [Rif] resistance detection was 87.5% and 81.82% respectively, while specificity was 100% for both pulmonary and extra-pulmonary TB. On comparison to sequencing of *rpoB* gene [Rif resistance determining region, RRDR], sensitivity was respectively 93.33% and 90% while specificity was 100% in both pulmonary and extra-pulmonary TB. GeneXpert assay missed 533CCG mutation in one sputum and dual mutation [517 & 519] in one pus sample, detected by sequencing. Sequencing picked dual mutation [529, 530] in a sputum sample sensitive to Rif, demonstrating, not all RRDR mutations lead to resistance.

**Conclusions:**

Current study reports observations in a patient care setting in a high burden region, from a large collection of pulmonary and extra-pulmonary samples and puts to rest questions regarding sensitivity, specificity, detection of infrequent mutations and mutations responsible for low-level Rif resistance by GeneXpert. Improvements in the assay could offer further improvement in sensitivity of detection in different patient samples; nevertheless it may be difficult to improve sensitivity of Rif resistance detection if only one gene is targeted. Assay specificity was high both for TB detection and Rif resistance detection. Despite a few misses, the assay offers major boost to early diagnosis of TB and MDR-TB, in difficult to diagnose pauci-bacillary TB.

## Introduction

In the absence of an efficient diagnostic modality for Tuberculosis [TB], the search for a tool that can overcome the dilemmas of the available diagnostic tests continues. In the wake of continued reports of high mortality and morbidity due to TB, WHO approved GeneXpert MTB/RIF [Cepheid, Sunnyvale, CA, USA] in 2011 and recommended it for rapid implementation [1]. Several studies have since published evaluation and validation reports [2, 3, 4].

TB control programs and treatment protocols primarily target active TB disease. Hence early diagnosis and institution of early treatment is not directed only towards the individual but also serves to prevent transmission in the community, especially important for drug resistant TB. Smear with its poor sensitivity and culture with the prolonged turnaround time fail to cater to this important need for early diagnosis. Gandhi et al reported that in a cohort of HIV-MDR-TB patients, 50% died before the culture/drug susceptibility testing [DST] report was generated [5]. It is towards this goal that rapid molecular diagnostics offering great promise have been developed [6].

The GeneXpert MTB/RIF System offers an efficient and rapid, near patient technology, capable of simultaneously detecting *M*. *tuberculosis* [MTB] and resistance to Rif. The hands-free sample processing and DNA extraction platform coupled with a Real-time PCR gives a Limit of Detection [LOD] of 4.5 genomes per reaction, and a clinical LOD of 131cfu/ml [2]. The reported sensitivity ranges between 72.5–98.2% [smear negative and smear positive samples respectively] with a specificity of 98.2% [3]. The system is easy to use, bio-safe and ensures absence of any sample cross contamination.

The limitations of the test include detection of drug resistance to Rif alone [hence its inability to identify XDR-TB from MDR-TB, [Rif being a surrogate marker for MDR]] and identification of drug resistance from a mixed population [the *rpoB* allele responsible for RIF resistance should be present in at least 65% of DNA present in the sample] [4]. Further limitations have been reported with respect to reporting of Leu533Pro mutations unless 100% of DNA population in the sample has the mutation [4, 7] an error that is reported to be overcome by using G4 cartridges [7].

The estimate of primary MDR-TB [MDR in new TB cases] in India is 2.2% while MDR-TB in re-treatment TB cases is 15% [8]. However studies from tertiary care centers report higher MDR-TB possibly due to referral bias and difficulty in diagnosing an indolent disease [9]. TB in EP sites is also a diagnosis more often confirmed in tertiary care centers due to complex clinical presentation and need for invasively collected samples. Currently the Revised National TB Control Program [RNTCP] endorses GeneXpert MTB/RIF for diagnosis of MDR TB among MDRTB suspects and for diagnosis of TB among certain vulnerable populations [HIV infected TB suspects and pediatric TB suspects] [10,11].

A few studies have raised concerns regarding the assay performance in patient care settings with respect to sensitivity, specificity, indeterminate results, inefficient detection of Rif mono-resistance and certain disputed mutations. The aim of the present study was to assess the performance of GeneXpert MTB/RIF for the diagnosis of pulmonary and extrapulmonary TB, and for the detection of Rif resistance in a tertiary level patient care setting

## Material and Methods

### Design, setting and study population

A prospective study was conducted at the Tuberculosis Division, Department of Microbiology, All India Institute of Medical Sciences New Delhi, India a multi-specialty tertiary care, teaching and referral hospital, with 2500 bed capacity. A number of patients with chronic extra-pulmonary presentations report to different specialties, as they remain undiagnosed in the peripheral centers. From March 2012 to June 2014, two thousand forty two patients presenting with symptoms potentially due to pulmonary or extra-pulmonary tuberculosis were screened and enrolled if they fulfilled the inclusion criteria. Patients with the following inclusion criteria were included in the study: Patients of either sex aged between 15 to 60 yrs, free from any underlying disease or prior infection of lung, anti-tuberculosis treatment [ATT] naïve, willing and able to give valid informed written consent. Patients with the following exclusion criteria were excluded from the study: patient unwilling and unable to give valid informed written consent, patients with known clinical diagnosis other than the disease in question, patients already on ATT, and patients with HIV infection.

**Fig 1:**
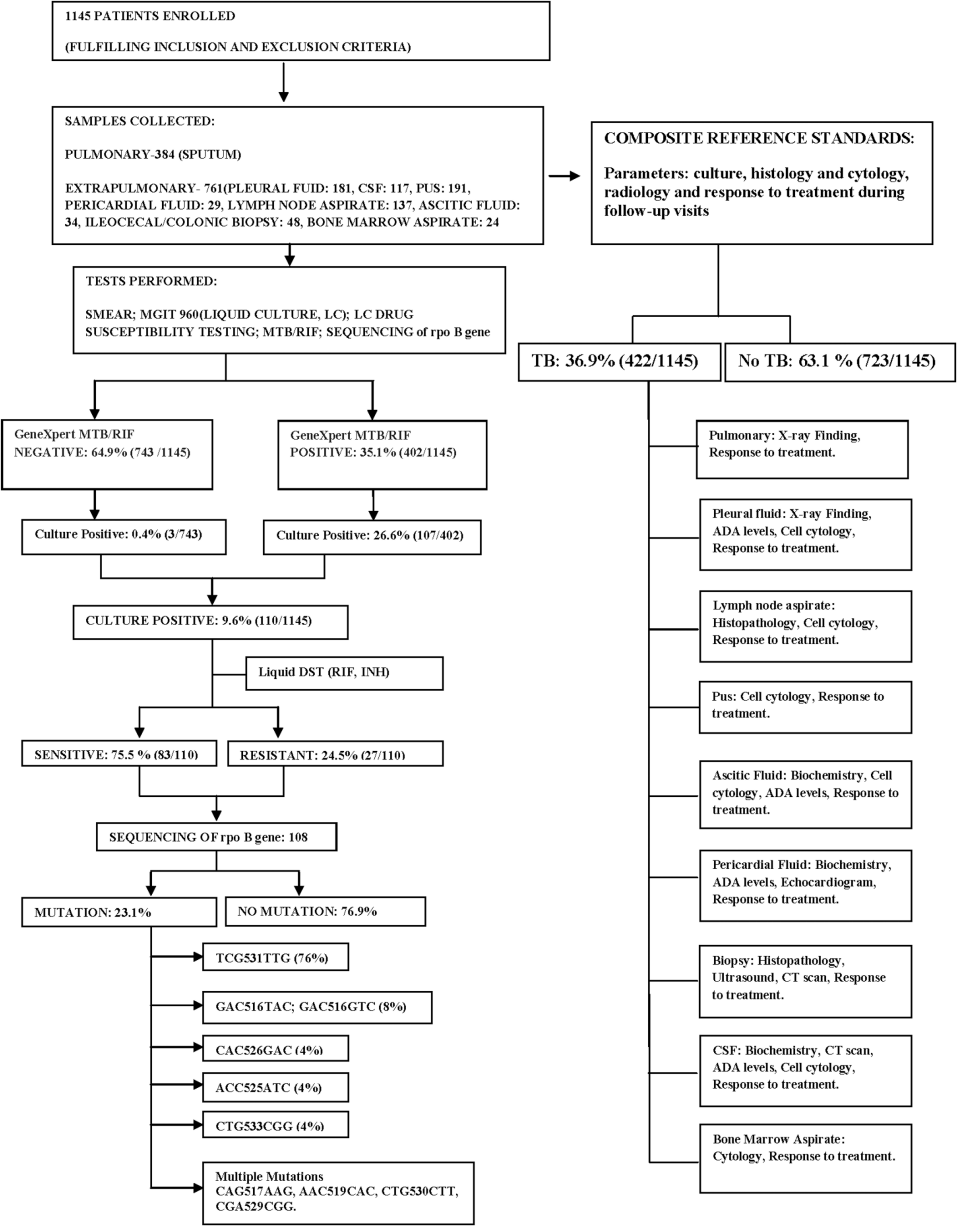
Study flow diagram. Diagnostic workflow of the patients included in the study.

One thousand one hundred forty five patients with high clinical suspicion of TB but not initiated on ATT at the time of enrollment, were enrolled in the study [Fig 1]. All samples were collected using standard protocol [12]. CSF was collected from patients presenting with fever, irritability, restlessness, neck stiffness and rigidity, headache persistent for 2–3 weeks, vomiting, seizures, and focal neurological deficit. Patients presenting with sub-acute intestinal obstruction, abdominal pain, alternating diarrhea and constipation were subjected to endoscopic/colonoscopic examination and biopsy from any abnormal/ulcerated region was collected for diagnostic workup. Patients presenting with enlargement of lymph nodes, neck swelling / draining sinus were subjected to “Fine needle aspiration” of the lymph nodes which was sent to the laboratory for the diagnostic tests. Patients presenting with shortness of breath, chest pain, dyspnea with fluid level and no other chest findings on the X-ray, were subjected to a pleural tap. The pleural fluid was further processed for smear, culture, and GeneXpert. Only one sample was collected where invasive techniques were used, while sputum samples were collected on two occasions from patients presenting with chronic cough, fever, chest pain, symptoms suggestive of pulmonary TB. Samples were divided into 3 aliquots namely for Smear microscopy and culture [LJ and MGIT 960 system], GeneXpert Assay and storage in -80^0^ C.

### Samples

The invasively collected specimens were processed singly. The sputum specimens were processed using NALC-NaOH method [N-acetyl-L-cysteine–NaOH–sodium citrate method] for making smears, and pooled for culture and GeneXpert tests [13]. Sterile body fluids were centrifuged and the pellet was used as inoculum.

### AFB smears

The processed specimens were used for making smears for all samples. All the smears were stained by the Ziehl Neelsen method and examined with a light microscope [14].

### MGIT 960

Samples were inoculated into MGIT 960 non-radiometric automated isolation system [Becton Dickinson, Sparks, MD, USA]; the MGIT tube containing 7 ml of 7H9 medium, supplemented with 0.8 ml of Oleic Acid-Albumin-Dextrose-Catalase [OADC] along with Polymyxin B- Amphotericin B—Nalidixic acid- Trimethoprim–Azlocillin [PANTA], was inoculated with 0.5 ml of decontaminated sample. Positive cultures were confirmed using TBc Identification Test [TBc ID, Becton Dickinson, Sparks, MD, USA] as *Mycobacterium tuberculosis* [15].

### DST

Drug susceptibility testing [DST] for Rif and INH was performed with the MGIT 960 system, using the WHO recommended standard critical concentration of 1 μg/ml Rif and 0.1 μg/ml INH. Standard protocol was followed according to the manufacturer’s instructions [15].

### GeneXpert

The Xpert assay was performed according to manufacturer’s instructions [Cepheid, Sunnyvale, CA] using latest version of G4 cartridges. [3,4,16]. In the Xpert assay, sample reagent was added at a 2:1 ratio to clinical specimens and it was incubated for 15 min at room temperature with intermittent shaking. Following which, 3 ml of the inactivated material was transferred to the cartridge. The cartridges were inserted into the test device and the results were generated after 90 min. [16] Repeat GeneXpert MTB/RIF tests were run for results that were indeterminate. GeneXpert results were interpreted by an independent observer without the knowledge of results of reference standard.

### Sequencing

The genomic bacterial DNA of culture isolates was obtained by heat lysis method [17] and amplified using standardized protocols. The 81-bp *rpoB* hot-spot region [RRDR]]region was amplified by PCR and DNA sequencing done with specific primers. For mutation analysis of the RRDR, a 437-bp fragment of the *rpoB* gene was amplified using primers

*rpoB*-for 5´ TGGTCCGCTTGCACGAGGGTCAGA-3´ and

*rpoB*-rev 5´-CTCAGGGGTTTCGATCGGGCACAT-3´ as described previously [18].

The cycling conditions for *rpo* B gene PCR were briefly, 35 cycles of 94°C for 1 min, 60°C for 1 min, and 72°C for 2 min. An aliquot [5μl] of PCR product was analysed by gel electrophoresis in 1.5% agarose gels. Cycle sequencing was performed using BigDye Terminator Ready Reaction Cycle Sequencing Kit [PE Biosystems, Foster City, Calif.] and the products were loaded in the ABI Prism 310 Genetic Analyzer [PE Biosystems], as instructed by the manufacturer. DNA sequences thus obtained were aligned for homology using Basic Local Alignment Search Tool algorithm in the Genbank database and analyzed for mutations with Genedoc Multiple Sequence Alignment Editor. [http://www.psc.edu/biomed/genedoc]. Sequences thus obtained were submitted to the Genbank and Accession numbers obtained [Genbank Accession Numbers KP658669-658720].

### Definitions

The patients were followed for two years following the diagnosis and institution of treatment. Adequate response to treatment was assessed in terms of improvement in signs and symptoms such as fever, lymphadenopathy, fluid collection, improvement in general well-being, and weight gain. The CRS for biopsy samples and aspirates included parameters such as culture, histology and cytology, radiology and response to treatment during follow-up visits. For sterile body fluids [pleural fluid, ascitic fluid, pericardial fluid and cerebrospinal fluid] in addition, biochemical tests included adenosine deaminase [ADA] levels.

### Study definitions

A case of pulmonary TB is considered to be smear-positive if one or more sputum smear specimens at the start of treatment are positive for AFB. New sputum smear-positive pulmonary TB case is the presence of at least one acid-fast bacillus [AFB+] in at least one sputum sample and a case of pulmonary TB is considered to be smear-negative if at least two sputum specimens at the start of treatment are negative for AFB in countries with a functional EQA system. [19]

### Reference standard comparison

The Xpert results from all the pulmonary and extra-pulmonary samples were compared with CRS as shown in Table 1. For pulmonary TB, diagnosis was given if any two of smear/culture/response to treatment/radiological findings were positive and for EPTB, diagnosis was given if any two of smear/culture/histopathology/cytology/biochemical analysis/ response to treatment/ADA levels/radiological findings were positive [Table 1]. Response to treatment was recorded at 6 months and again at 2 years follow-up.

**Table 1:** Performance of the GeneXpert for the diagnosis of TB: Sensitivity, Specificity of GeneXpert MTB/RIF in comparison with Composite Reference Standards.

	**GENEXPERT**	**COMPOSITE REFERENCE STANDARDS ^a^ (6 month follow-up)**		**COMPOSITE REFERENCE STANDARDS ^b^ (2 yr follow-up)**	
		**TB**	**NO TB**	**TB**	**NO TB**
	POSITIVE (186)	178	8	186	0
	NEGATIVE (198)	0	198	0	198
	SENSITIVITY	100%(95% C.I: 97.95% to 100.00%)		100% (95% C.I: 98.04% to 100.00%)	
	SPECIFICITY	96.1%(95% C.I: 92.5% to 98.3%)		100% (95% C.I: 98.15% to 100.00%)	
	**GENEXPERT**	**COMPOSITE REFERENCE STANDARDS ^c^ (6 month follow-up)**		**COMPOSITE REFERENCE STANDARDS ^d^ (2 yr follow-up)**	
**EXTRA-PULMONARY SAMPLES (n = 761)**	**GENEXPERT**	**TB**	**NO TB**	**TB**	**NOTB**
	POSITIVE (216)	168	48	214	2
	NEGATIVE (545)	22	523	22	523
	SENSITIVITY	88.42%(95% C.I: 83.52% to 92.99%)		90.68%(95% C.I: 86.23% to 94.07%)	
	SPECIFICITY	91.59%(95% C.I: 89.01% to 93.74%)		99.62%(95% C.I: 98.63% to 99.95%)	

Data are presented as percentage at 95% CI. All pulmonary and extra-pulmonary samples in the study are included in the analysis.

^a^ For patients with suspicion of Pulmonary TB, diagnosis of TB was given if any two of smear/culture/response to treatment at 6 months/radiological findings were positive.

^b^ For patients with suspicion of Pulmonary TB, diagnosis of TB was given if any two of smear/culture/response to treatment at 2 years/radiological findings were positive.

^c^ For patients with suspicion of Extra-pulmonary TB, diagnosis of TB was given if any two of smear/ culture/ histopathology/ cytology/biochemical analysis/ response to treatment at 6 months/ADA levels/radiological findings were positive].

^d^ For patients with suspicion of Extra-pulmonary TB, diagnosis of TB was given if any two of smear/ culture/ histopathology/ cytology/biochemical analysis/ response to treatment at 2 years/ADA levels/radiological findings were positive.

Culture [including Liquid Culture [LC]] is an accepted gold standard. [20] Nevertheless, in pauci-bacillary disease such as EPTB, the culture yield may be poor, while the molecular tests have the ability to detect DNA from non-viable organisms with a LOD ranging between 5 to 100 bacilli/ml, hence may detect culture negative samples. CRS has been used as gold standard in studies to overcome such issues, though it may suffer from poor specificity. [21] Hence, both culture and CRS were used as reference standards in order to reach an optimum sensitivity and specificity [Table 1]. In addition sequencing was used to identify the mutations for comparison with GeneXpert results [Table 2].

**Table 2:** Performance of Genexpert for detection of RIF resistance: sensitivity and specificity of the genexpert for detection of RIF resistance in comparison with gold standard phenotypic [MGIT 960, LC-DST] and genotypic [sequencing] results.

		MTB drug resistance detection (Gold standard methods)			
SAMPLES	MTB drug resistance detection by Xpert (n = 72)	Phenotypic method Culture-DST (MGIT960)		Genotypic method *rpoB* sequencing (RRDR)	
**PULMONARY SAMPLES**		Resistant	Sensitive	Resistant	Sensitive
	Resistant	14	0	14	0
	Sensitive	2	56	1	57
	Sensitivity	87.5% [95%CI:61.65% to 98.45%]		93.33%[95%CI: 68.05% to 99.83%]	
	Specificity	100%[95% CI: 93.62% to 100.00%]		100%[95% CI: 93.62% to 100.0%]	
**EXTRA-PULMONARY SAMPLES**	MTB drug resistance detection by Xpert (n = 35)	Phenotypic method Culture-DST (MGIT960)		Genotypic method *rpoB* sequencing (RRDR)	
		Resistant	Sensitive	Resistant	Sensitive
	Resistant	9	0	9	0
	Sensitive	2	24	1	25
	Sensitivity	81.82% [95%CI: 48.22% to 97.72%]		90% [95%CI: 55.50% to 98.75%]	
	Specificity	100% [95%CI: 85.75% to 100.00%]		100% [95%CI: 86.28% to 100.00%]	

### Statistical analysis

The sample size was calculated prior to the initiation of the study. The sample size was established using the Statistical formula:

N=Z2P[1−P]/d2

Where P = prevalence and d = precision, using the test sensitivity as 80% and specificity 98% [3]. P is pre-determined value of sensitivity [or specificity] that is ascertained by previous published data or clinician experience/judgment. A sample size of 693 was suggested for EPTB including controls.

Sensitivity and specificity, of the GeneXpert MTB/RIF, respective 95% confidence intervals [95% CI] were calculated after comparison to the results of the phenotypic gold standard MGIT 960, [liquid culture drug susceptibility testing LC-DST] and CRS.

Kappa chi square test was performed for the agreement analysis. A p-value of 0.05 was considered as statistically significant. All data were analyzed using STATA statistical software version 12.1 [StataCorp LP, College Station, TX, USA].

### Forest plot

Forest plot was drawn in order to draw comparison with available published literature on the use of GeneXpert in EPTB. For each published study, we calculated GeneXpert MTB/RIF sensitivity and specificity along with 95% confidence intervals, compared with CRS, and generated forest plots to display sensitivity and specificity estimates using STATA statistical software version 11. The sensitivity and specificity varied across the samples. The square boxes indicate the size of the study. The dotted line is a visual assessment of the heterogeneity of the studies. Confidence Intervals are a measure of the precision of the results of a study.

### Ethics

The study was approved by the AIIMS Ethics Committee [IEC/NP-105/2011& RP-16/2012]. A written consent was obtained from all the participants.

## Results

### Characteristics of the study population

Patients enrolled for the study were in the male: female ratio of 1.06. There were more males in the 15–30 years age group [ratio 1.15] and almost equal in number in 31–60 years age group [1.02]. Samples collected for the study included Sputum: 384, Pleural fluid: 181, Pus: 191, lymph nodes aspirate: 137, Cerebrospinal Fluid [CSF]: 117, Ascitic fluid: 34, Pericardial fluid: 29, Bone marrow aspirate: 24.

### Performance of GeneXpert for the diagnosis of TB

In pulmonary group GeneXpert detected TB in 72 culture positive and 114 culture negative patients, while in extra-pulmonary group it detected TB in 35 culture positive and 181 culture negative patients, thus aiding treatment decisions in 295 patients in whom culture was negative.

### Comparison of results with composite reference standards

An interesting finding [Table 1] was a group of patients who were GeneXpert test positive but for whom a delayed/ no response to treatment was recorded at 6 months. Forty-one of 48 such patients with EPTB were resistant to Rifampicin by GeneXpert and were given treatment for MDR-TB, while four had gastrointestinal TB, and three had CNS-TB. Two-year follow-up showed response to treatment in all the 48 MDR-TB patients except two with CNS-TB.

### Indeterminate results

Four samples [4/1145] were flagged as ‘*M*. *tuberculosis* detected, very low, Rif resistance Indeterminate’. Cultures were negative in ¾ samples, possibly due to low bacillary load. Results were concluded on repeating GeneXpert test [for two extra-pulmonary samples] and repeating samples [for two sputum samples]; Rif resistance in one [sputum], Rif sensitive in two [sputum, pus] and ‘*M*. *tuberculosis* not detected’ in one [lymph node aspirate]. Indeterminate results were seen only in ‘Very low’ bacillary load.

### Performance of GeneXpert for detection of Rif resistance

**Fig 2:**
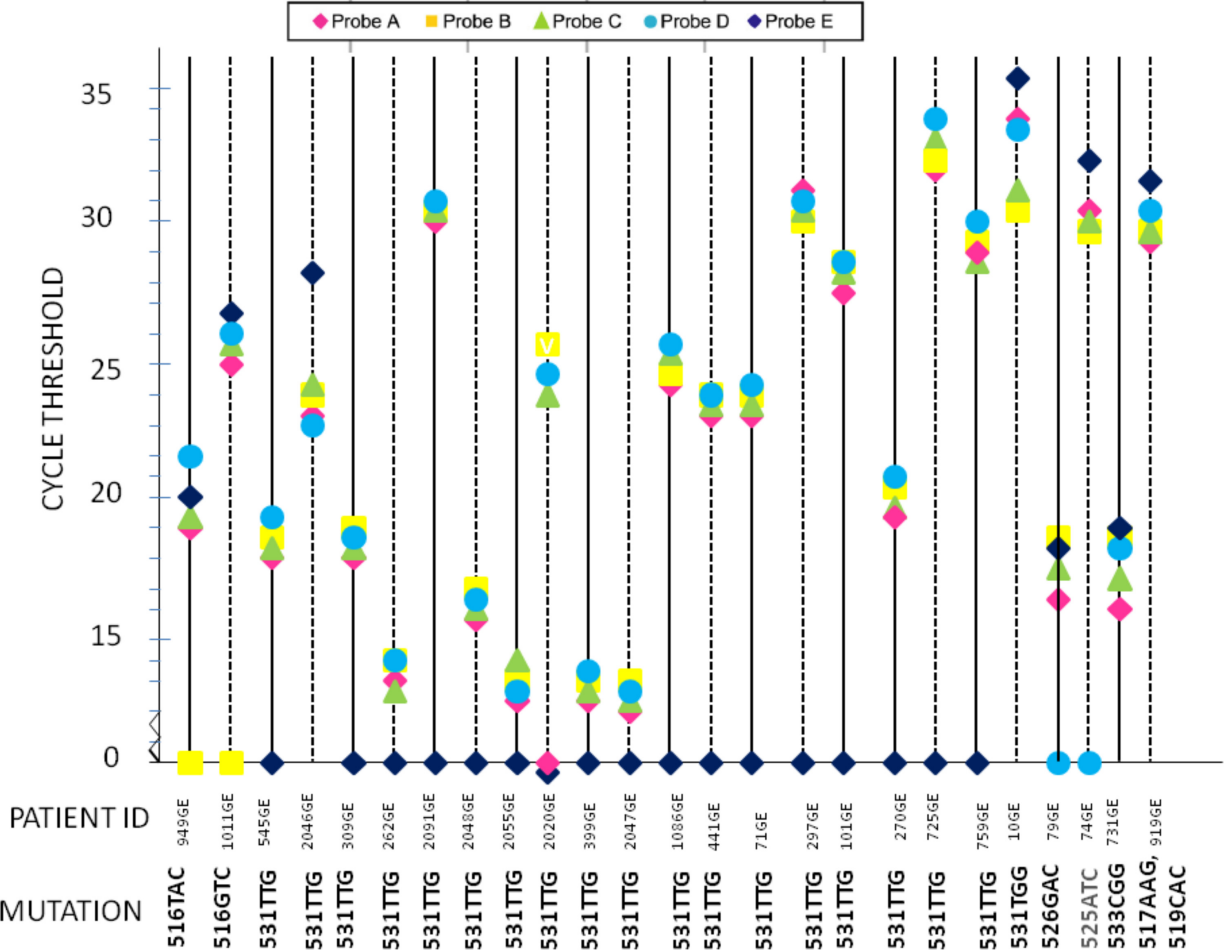
GeneXpert Detection of mutations in *rpoB* RRDR region. Results from twenty-five Rif resistant samples are shown. The results produced by each sample are indicated by a single vertical line on which the C_T_ value of each of the five *rpoB*-specific molecular beacons [probes A to E] is plotted. Twenty-three samples were correctly identified as Rif resistant. Two samples [731GE, 919GE] were missed by GeneXpert but showed mutations in RRDR region on *rpoB* gene Sequencing [533CGG, 517AAG and 519CAC respectively] [Genbank Accession Numbers KP658669-658720]. Two strains with 531TTG mutation were detected owing to difference between the C_T_ value being >4 and not due to loss of probe.

Of the culture positive samples, the Xpert MTB/RIF assay detected Rif resistance in 14/72 pulmonary samples and 9/35 extra-pulmonary samples. The *C*_*T*_ values for different probes for the Rif resistant samples are plotted in Fig 2. Twenty-three samples were correctly identified as Rif resistant. Twenty-one of 23 Rif resistant samples had at least one negative probe, while two samples were detected as resistant as the ΔCt was more than 4 between any 2 probes.

### Comparison of GeneXpert assay results with phenotypic and genotypic gold standards

Seventy-two pulmonary and 35 extra-pulmonary samples were culture positive. These were further subjected to DST on MGIT 960 [LC-DST] and PCR-sequencing. The results on LC-DST were compared to the Xpert MTB/RIF assay [Table 2] The DST results were discordant with the Xpert results in 2/72 pulmonary samples and 2/35 extra-pulmonary samples. In all the four cases the Xpert showed the sample as sensitive while the culture isolates were resistant [0.5ug/ml and 1ug/ml Rifampicin] [Table 3].

**Table 3:** Samples with Discordant Results.

Patient ID	*Mycobacterium tuberculosis* Genotypic drug resistance detection			
	0.5μg/ml	1 μg/ml	*Rpo* B Sequencing result [RRDR]	GeneXpert result
731 [SPUTUM]	RESISTANT	RESISTANT	CTG533CGG	MTB detected, Rifampicin sensitive.
1014 [SPUTUM]	RESISTANT	RESISTANT	NO MUTATION	MTB detected, Rifampicin sensitive.
30B[LYMPH NODE ASPIRATE]	RESISTANT	RESISTANT	NO MUTATION	MTB detected, Rifampicin sensitive.
919GE [PUS]	RESISTANT	RESISTANT	CAG517AAG AAC519CAC	MTB detected, Rifampicin sensitive.

Summary of MICs determined by LC-DST in the automated MGIT 960 system, GeneXpert results, rpoB gene Sequencing for five M. tuberculosis isolates. While four GeneXpert results were discordant with LC-DST, two of the isolates did not show any mutation in the rpoB RRDR region, indicating resistance due to sites outside RRDR. [Genbank Accession Numbers KP658669-658720]

Total resistant: 27

Discordant results between Genexpert & LC-DST: 4

Discordant results between Genexpert & sequencing: 2 [1 pulmonary,1 extra-pulmonary]

Discordance between the two gold standards: 2 [1 pulmonary,1 extra-pulmonary][due to restricting genotypic evaluation to RRDR]

Further, using sequencing as gold standard, the Rif resistance mutations were detected in 25/27 samples resistant to Rif by LC-DST [Table 2]. One pulmonary and one extra-pulmonary sample had been incorrectly missed by the Xpert assay [Table 2]. In addition, two samples missed by Xpert assay but resistant by LC-DST did not show any mutation in the RRDR region sequenced. The resistance in these two cases may be ascribed to other mechanisms such as efflux-pumps or mutations outside of RRDR. One isolate from sputum sample was sensitive to Rif by LC-DST, sensitive by Xpert but showed two mutations on sequencing, CGA529CGG, CTG530CTT.These mutations have not been reported or associated with drug resistance before, and may possibly be silent mutations.

### Bacillary load and drug resistance

Quantitation of bacillary load by Xpert assay is determined by threshold-cycle [Ct]; High Ct value is <16; Medium Ct value is16-22; Low Ct value is 22–28; Very Low Ct value is >28. The higher bacillary load, when plotted against Rif and INH resistance profiles showed a statistically significant association with drug resistance [Table 4].

**Table 4:** Correlation of Bacterial load [GeneXpert] with Drug susceptibility pattern [LC-DST].

Resistance pattern	Bacillary load				p value
PULMONARY SAMPLES	HIGH	MEDIUM	LOW	VERY LOW	
RIF RESISTANT(n = 16)	12	1	2	1	0.001
RIF SENSITIVE (n = 56)	2	3	17	34	
INH RESISTANT(n = 16)	10	1	2	2	0.001
INH SENSITIVE (n = 56)	3	3	16	34	
EXTRA-PULMONARY SAMPLES	HIGH	MEDIUM	LOW	VERY LOW	
RIF RESISTANT(n = 11)	3	3	5	0	0.010
RIF SENSITIVE (n = 23)	0	3	13	7	
INH RESISTANT(n = 12)	0	3	6	3	0.789
INH SENSITIVE (n = 23)	0	3	13	7	

Statistically significant association was found between the bacillary load as detected by GeneXpert system in pulmonary samples [p<0.001] and drug resistance both to Rif and INH, detected using LC-DST, however most of the extra-pulmonary samples were pauci-bacillary to draw any correct inference.

### Mutations identified by sequencing, missed by Xpert assay

The G4 cartridges have been improvised to address the detection of mutation at 533 CCG. Nevertheless, one culture with the 533 CCG mutation was missed in the study [Δ Ct was 3.1 between 2 probes, system is capable of detecting >4]. In addition the Xpert assay missed the mutations CAG517AAG, AAC519CAC in one isolate from pus sample detected resistant in LC-DST [Δ Ct was 2 between 2 probes], and CGA529CGG, CTG530CTT, in one isolate from sputum, sensitive in LC-DST [Δ Ct was 2.2 between 2 probes].

### Rifampicin mono-resistance, INH resistance

Two isolates showed resistance to Rif [1ug/ml] while being sensitive to INH [0.1ug/ml] by LC-DST in MGIT-960. Sequencing ascribed Rif resistance to 531 TTG in one sample [sputum] and 533CCG in the second sample [sputum, sensitive by Xpert assay]. INH resistance without Rif resistance was detected in three isolates.

## Discussion

The Xpert assay has brought about a major change in the speed, simplicity and accuracy of not only diagnosis of TB but also drug resistance to RIF in TB, which is accepted as a surrogate for MDR-TB. The sensitivity of detection enables diagnosis in smear negative and often culture negative TB. The rapidity and robustness of diagnosis in-turn breaks the chain of transmission in addition to early institution of treatment and improved chances for cure. The utility of Xpert assay in diagnosis of pauci-bacillary TB is the most important contribution of the test. WHO policy document 2013 adopted a GRADE system approach to arrive at recommendations [9] on the diagnostic value of the assay in Pulmonary and EPTB.

RIF resistance detection in the Xpert assay is based on hybridization [or the absence] of five molecular beacon probes complementary to the wild type sequence of *rpoB* gene [responsible for 95% of drug resistance mutations in the RRDR, codons 507 to 533]. The inherent nature of the technique being highly dependent on a strict ionic milieu and inter-molecular forces, may sometimes subject the fidelity to question. Blakemore et al 2012 [4] suggest that the detection of mutations in RRDR varies with the nature of the mutations; the mutations capable of completely inhibiting the binding of molecular beacons are easier to detect than the ones causing a delay. Most of the Rif resistance mutations are of the first kind hence are easily detected [2].

The use of wild type probes ensures the detection of rarely reported or unreported mutations as well. Some of the *rpoB* mutations have been reported to be associated with Rif susceptible genotypes [22–28]. The data on these issues are contradictory. The detection of only the RRDR mutations misses the resistance determining mutations outside the hotspot, such as 572Phe and a few others, together responsible for upto 5% of all Rif mutations [28], possibly responsible for two isolates in our study.

In the present study, Xpert detected four samples discordant with culture DST. On sequencing, two showed mutations [517,519 [dual mutation] and 533CCG], while two others had no mutation in the RRDR. Kim et al 2012 suggested that the mutations at probe ends might be missed [29]. In their study a Δ Ct of 3.2 was responsible for the failure of detection of 518Asp mutation by Probe C as it was lying at the probe end. The missed dual mutations in the present study, 517, 519 and 529,530 are at the junction of Probes B & C, and D & E, and may have been missed due to competition for binding.

The detection of 533Pro has long been a subject of debate till the time the G3 cartridges were in use; with the new G4 cartridges the error rate was expected to be low [23]. For detection by Xpert, 100% mutant DNA with 533 CCG mutation is required, while in case of 531 CTG mutations, 65% mutant DNA is adequate for detection [4].

Mixed MTB infections have been suggested to be responsible for false negative and positive results for RIF by Xpert [30]. Hetero-resistance defined as the presence of both sensitive and resistantMTB populations is often suggested to be responsible for discordant DST results [23]. Several studies have shown that mixed populations do not usually occur [2,31–32], however reports of mixed infections warrant further studies to evaluate its interference with drug resistance detection [33,34]. For the same reason, Xpert cannot be used for assessing the emergence of Rif resistance during treatment [4].

Line Probe Assay [LPA] is said to be capable of indicating hetero-resistance but the LOD of LPA is at least 5X10^3^ bacilli per ml of sample, hence the bacillary load in the mixed population would need to be high enough for detection using reverse hybridization by LPA. The LPA also may not detect all mutations at position 533; the probes are so designed that the mutation does not always affect the loss of binding of probes [35,36]. Chakravorty et al 2012 used ‘sloppy molecular beacons’ to enable detection of 40% mutant DNA in samples, equivalent to that detected by sequencing [37].The study demonstrated statistically significant correlation between Rif and INH resistance and the bacterial load as determined by the Ct value. This seconds the findings by several researchers who have demonstrated increased chances of drug resistance in higher bacillary loads. Higher bacillary load has been shown to predispose to accumulation of resistance mutations due to higher number of replication cycles and hence higher chances of occurrence and accumulation of such mutations [38].

The association of mutations at codon 531 and some substitutions at codon 526 [Tyrosine and Aspartic Acid] with High-level Resistance [HLR] and of certain other mutations with Low-level Rif Resistance [LLR], and susceptibility to Rifabutin [H526L, F514FF, D516V, S522L and mutations at codons 516, 529, and 533] could prove useful in treatment decisions in certain cases [24]. Possible inclusion of specific mutant probes in the GeneXpert test design could assist treatment decisions. The frequency of 533Pro mutation responsible for LLR has been reported globally to range from 3% to 6% [39] and hence may not be as salient as the other more frequent HLR mutations.

In the present study, GeneXpert assay was extremely useful in establishing an early diagnosis in several pulmonary and extra-pulmonary samples, in which the smears as well as culture failed to give any clue to diagnosis. The use of CRS offered a good comparison for GeneXpert. Few patients with EPTB, who had been diagnosed as resistant to Rif showed a delayed response, though on MDR-TB treatment. Response to treatment at such extra-pulmonary sites may take longer to appear. Two-year follow-up in these patients showed resolution of symptoms in most patients.

**Fig 3:**
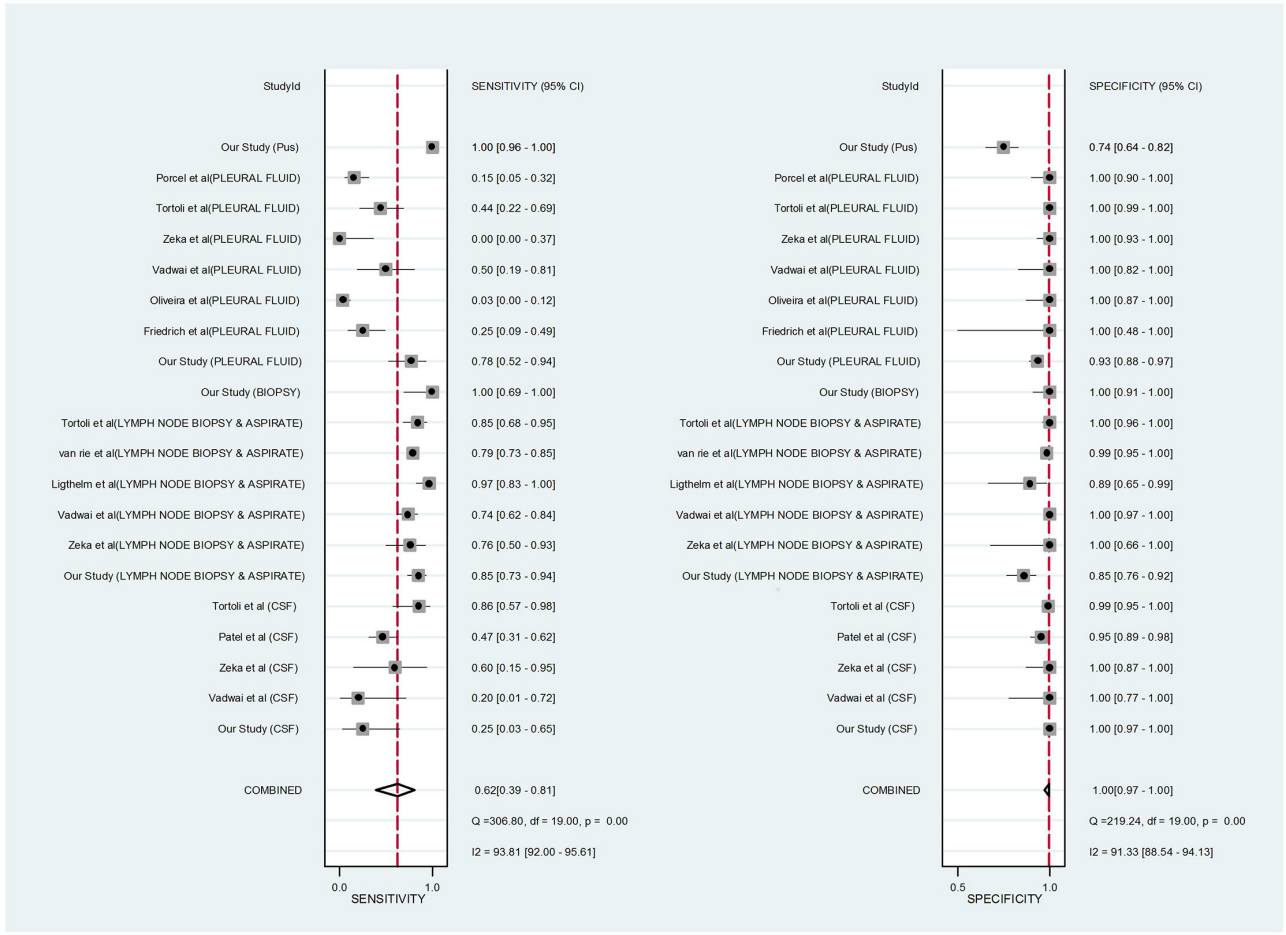
Forest plot showing Genexpert MTB/RIF sensitivity and specificity for tuberculosis detection against the composite reference standards, in different types of Extra-pulmonary samples when compared with other published studies. The squares represent the sensitivity and specificity; the black line indicates the confidence interval. [42–46]

Forest plot in Fig 3 compares the sensitivity and specificity for diagnosis of TB by GeneXpert in the present study with published work. The sensitivity for TB detection in CSF samples was similar to the study by Vadwai et al. while it was better than other studies in pleural fluid samples and lymph node aspirates. Specificity of TB detection was comparable to other studies for CSF samples, but slightly lower for other samples. Variations in different studies suggest optimization of sample processing protocols for foolproof amplification results. [Part of the study data contributed to the WHO policy document 2013, [obscured, awaiting publication] [8].

Van Deun et al 2013 and Jamieson et al 2014 have shown that cultures with certain ‘disputed’ drug resistance mutations [511Pro, 516Tyr, 526Asn, 526Leu, 533Pro, and 572Phe] may be slow to grow in the presence of drug [fitness loss] and hence may be labeled as sensitive and would be ideally identified from the sample directly [24, 28] In our series both MGIT and Xpert detected all so-called ‘disputed’ mutations except one 533CCG mutation. However several studies have demonstrated the need to revise the critical concentrations used in MGIT-DST [Phenotypic gold standard] from 1ug/ml to 0.5ug/ml or even lower [24, 28, 40, 41].

The relevance of all the tests and efforts at resistance detection should translate into clinical care, predict the drugs useful and those not useful. Rif is the mainstay of TB treatment, removal of Rif from treatment protocol is a major decision. Phenotypic DST based on culture, though slow, detects all clinically relevant resistance and clearly serves as the gold standard for drug resistance diagnosis. Including one extra tube with 0.5ug/ml Rif can easily incorporate the detection of mutations responsible for low-level resistance to Rif. The drug levels achievable with 600 mg dosage of Rif is 1.5ug/ml of free drug, which is capable of inactivating strains with MIC up to 0.375ug/ml. Therapeutic drug monitoring can further assist treatment design.

Genotypic assays though offer rapidity and most often a good sensitivity when the probes designed can cover all possible mutations responsible for resistance but could give false positive results due to detection of mutations not responsible for resistance. Also the current methods cannot detect the level of drug resistance, hence cannot be used for deciding in favor of Rifabutin as an alternative drug of choice. The current design of the Xpert assay would miss the resistance detection in mixed samples and Rif resistance outside RRDR. Bacterial load in the samples may have a bearing on the resistance detection; hence repeat sampling may not always resolve the issue of resistance in low load samples. Nevertheless Xpert is the best bet today that offers rapid detection of Rif resistance with reasonable precision. It is rightly recommended to be the “Initial Diagnostic test”, aided by conventional microscopy and culture for monitoring therapy and DST for INH and second-line drugs especially in Rif resistant cases.
